# Age-related delay in information accrual for faces: Evidence from a parametric, single-trial EEG approach

**DOI:** 10.1186/1471-2202-10-114

**Published:** 2009-09-09

**Authors:** Guillaume A Rousselet, Jesse S Husk, Cyril R Pernet, Carl M Gaspar, Patrick J Bennett, Allison B Sekuler

**Affiliations:** 1 Centre for Cognitive Neuroimaging (CCNi) and Department of Psychology, University of Glasgow, Glasgow, UK; 2 McMaster University, Department of Psychology, Behaviour & Neuroscience, Hamilton, ON, Canada; 3 SFC Brain Imaging Research Centre, Division of Clinical Neurosciences, Western General Hospital, Edinburgh, UK

## Abstract

**Background:**

In this study, we quantified age-related changes in the time-course of face processing by means of an innovative single-trial ERP approach. Unlike analyses used in previous studies, our approach does not rely on peak measurements and can provide a more sensitive measure of processing delays. Young and old adults (mean ages 22 and 70 years) performed a non-speeded discrimination task between two faces. The phase spectrum of these faces was manipulated parametrically to create pictures that ranged between pure noise (0% phase information) and the undistorted signal (100% phase information), with five intermediate steps.

**Results:**

Behavioural 75% correct thresholds were on average lower, and maximum accuracy was higher, in younger than older observers. ERPs from each subject were entered into a single-trial general linear regression model to identify variations in neural activity statistically associated with changes in image structure. The earliest age-related ERP differences occurred in the time window of the N170. Older observers had a significantly stronger N170 in response to noise, but this age difference decreased with increasing phase information. Overall, manipulating image phase information had a greater effect on ERPs from younger observers, which was quantified using a hierarchical modelling approach. Importantly, visual activity was modulated by the same stimulus parameters in younger and older subjects. The fit of the model, indexed by R^2^, was computed at multiple post-stimulus time points. The time-course of the R^2 ^function showed a significantly slower processing in older observers starting around 120 ms after stimulus onset. This age-related delay increased over time to reach a maximum around 190 ms, at which latency younger observers had around 50 ms time lead over older observers.

**Conclusion:**

Using a component-free ERP analysis that provides a precise timing of the visual system sensitivity to image structure, the current study demonstrates that older observers accumulate face information more slowly than younger subjects. Additionally, the N170 appears to be less face-sensitive in older observers.

## Background

Ageing has widespread effects on visual functions, both in terms of scale, from cellular to behavioural changes, and in terms of areas affected, from the structural integrity of the eye to the frontal cortex [1-3]. However, despite changes in optical factors in the retina, and in the lateral geniculate nuclei of the thalamus (LGN), declines in visual functions with age are mediated, to a large extent, by cortical changes [1,4-6]. At the moment, we have a very poor understanding of age-related changes in visual cortical information processing. Although age-related changes in lower level vision, such as acuity and contrast sensitivity, are well documented [1,7], the study of higher-order brain processes, such as object recognition and attention remains in its infancy [6]. In humans, there is evidence that ageing affects a large range of visual processing tasks [6-8], including orientation discrimination [9], motion perception [10,11], contour integration [12], and face and object visual processing [13-16]. However, which stages of object visual processing are affected by ageing is still a controversial issue. Indeed, the part of the human brain that is devoted to object processing is distributed and essentially hierarchical in nature, with object information extracted progressively from the retinal input onward [17]. This functional organisation opens the possibility that age-related changes could impact different nodes of the object network. This question is important because ageing does not have a uniform effect on the brain. Rather, different brain areas undergo different anatomical and physiological changes at different rates, thus leading to stronger deficits in some tasks and brain functions than others [1,3,18].

In the primary visual cortex, V1, no systematic loss of neurons has been reported. However, a degradation of the receptive field properties of cortical neurons, higher spontaneous and evoked activities, and lower signal to noise ratio have been observed in monkeys [4,19]. In addition, structural changes related to a degradation of myelinated fibres, dendrites, and synapses have been described [20-22]. Importantly, age-related slowing of information processing has been observed in the primary visual cortex but not in the LGN [4]. Slowing in visual information processing could be due to decreased signal to noise ratios and decreased selectivity in V1 [19,23] and V2 [24], leading to a longer accumulation of information before a decision threshold can be reached. Myelin alterations could also be directly responsible for this age-related slowing of visual processing [18,20]. Overall, both structural and physiological evidence in monkeys suggest that the whole cascade of information processing, along the occipital-temporal pathway involved in object processing, might be perturbed by senescence.

So far, studies performed in humans using imaging techniques have failed to corroborate this prediction, and have provided heterogeneous results, whether they used non-face stimuli or face stimuli.

### ERP studies of visual ageing: non-face stimuli

In humans, we can infer the timing of information processing using measures of brain activity such as EEG or MEG. The evoked electrical visual activity, termed ERP (Event-Related Potential), is the most frequently used dependent variable to assess age-related changes in visual processing speed [25]. There is ample evidence that within 200 ms the entire visual pathways have been activated, allowing time for iterative interactions between distant cortical areas, even though not all perceptual tasks might be completed in 200 ms [26-28].

Using simple stimuli like checkerboard patterns, earlier studies have reported a consistent age-related delay in the P1 (P100) component that peaks around 80-120 ms [1,29], and has sources in the extra-striate cortex [27]. However, the effect of ageing was reduced or even absent in certain studies using stimuli at high-contrast, high-luminance, lower spatial frequencies [1,29], and for achromatic, rather than chromatic, horizontal sine wave gratings [30]. Even in studies reporting age-related delays in ERP components, it is striking to see that despite the global age-group effects, there tends to be a substantial overlap between younger and older subjects. Why some older subjects have results similar to those of younger subjects remains to be explained.

Other recent studies have failed to find age-related slowing as indexed by the latency of ERP components. For instance, a study using arrowheads found no significant increase in the latency of the P1 and the N1 components with age [31]. The N1 is typically recorded around 130-200 ms after stimulus onset and has distributed sources in the ventral and dorsal pathways [27]. Similarly, no age effect was found on P1 and N1 latencies evoked by triangular light flashes [32], and no change in an early frontal component (50-75 ms) was observed in response to line drawings [33]. Other studies have reported dissociations among different time windows. For instance, in response to circles and squares presented at 6.5° from a central fixation point, no age-related change was observed in the latency of the contralateral P1 (~92-96 ms). However, there were significant age-related increases in latency for the ipsilateral P1 (~120-136 ms) and the N1 [34], suggesting an age-related delay in interhemispheric communication, potentially mediated by impaired myelin. Conversely, age-related increases in peak latency have been reported in early but not later components, e.g. delay in P1 but not in N1 component [35-37]. Importantly, some early age-related changes are task dependent, which is consistent with the finding that early visual evoked activity can be modulated by task factors [36,38,39]. Therefore, the lack of consistency observed in the ageing literature considered so far might be related to differences in stimuli and task constraints.

### ERP studies of visual ageing: face stimuli

Because of their importance in social interactions, and because we have so much experience interacting with them, faces might constitute good stimuli to assess age-related changes in visual functions. Also, because large cortical evoked responses to faces are found in almost all subjects, using face stimuli might make it easier to compare results across ageing studies.

Few ERP studies have directly investigated age-related changes in the time-course of face processing. Three studies have reported an effect of ageing only on relatively late stages (>200 ms) of visual processing, thus suggesting that ageing spares early perceptual processes [40-42], while results from two other studies indicate an increase in the latency of the N170 [39,43], and in the latency of the M170 [44]. The N170 is an occipital-temporal component that tends to be larger in response to faces compared to other objects [25,45-49], and seems to have multiple cortical sources, including ventral (fusiform gyrus), and occipital-temporal areas [50-53].

The M170 is a magnetic component evoked by faces in the same time window as the N170, and the two components might originate from partially overlapping cortical sources [51,52]. In [44], no age difference was observed in the latency of the M100, peaking in the same time window as the EEG P1. Finally, two more recent EEG studies did report significant age-related N170 latency increases, also in the absence of P1 latency effects [39,43].

Thus, three out of six studies on age-related changes in face processing speed performed so far have identified the N170 as the first component that exhibits longer latency with increasing age. The reason for the absence of an effect in the three other studies remains unexplained. All six studies used different tasks, stimuli, and slightly different age groups, so the difference among studies might originate from a combination of these factors. More importantly, these studies suffer from methodological problems that limit their capacity to measure age-related changes in processing speed. We propose a different approach in an attempt to overcome these limitations.

### Present ERP experiment

Previous ERP studies focused their analyses entirely on peaks of various components of the evoked response, under the assumption that they reflect interesting neural processes (e.g., that the N170 ERP component reflects the encoding of faces). However, peaks are ill-defined in terms of selectivity to categories and processes [54,55]. Also, peaks can be difficult to measure in the presence of noise, for instance when noisy stimuli trigger weak evoked-responses. Previous studies also used mean ERP data, thus discarding the potentially rich variation in information processing that occurs across single-trials [56]. Furthermore, statistical analyses were performed at the group level only, without providing an assessment of visual processing speed in each subject individually. Finally, when significant effects were reported, the degree of overlap between age groups was not mentioned, except in one study, in which scatter plots of the data revealed both a mean latency increase with age, but also an increase in variance and an overlap among age groups [44], similar to results obtained using more simple stimuli [1,29]. Therefore, because of measurements limited to peaks and the lack of descriptive statistics, it is difficult to quantify the size of the age effects on the time course of visual processing found in previous studies.

Inspired by a recent change of philosophy in ERP research [54,55,57-59], Rousselet et al. (2008) [60] proposed an alternative approach to study visual face processing speed. Our approach uses parametric manipulations of visual stimuli that are designed to make it easier to identify the stimulus information that affects the activity of the visual system, as reflected by modulations of the EEG signal. Specifically, we manipulated image structure systematically by varying the phase spectra of our visual stimuli. Because the phase of an image's Fourier components carries much of the information about object identity [61,62], we degraded the natural appearance of face stimuli by manipulating image phase along a single continuum from original phase (maximal coherence) to completely random phase (no coherence). The use of a parametric design allows the expression of time-resolved brain activity, like EEG signals, in terms of sensitivity to stimulus information rather than signal amplitude. In other words, it allows us to measure the noise tuning function of the early EEG activity evoked by faces. This approach is applied over the whole data space (i.e., at all electrodes and time points), thus providing a spatial-temporal mapping of information processing that is not constrained to predefined time-windows.

Data processing follows a hierarchical procedure. A first round of analyses is performed on each subject individually, using a single-trial linear regression model, so that information processing speed can be determined for each subject. The linear regression model includes stimulus factors like phase coherence and other image metrics that are related to phase coherence. In other words, we identify variations in neural activity across trials that are statistically associated with changes to visual information. Strong associations at certain time-points imply that the visual system activity is significantly modulated by image characteristics. A second round of analyses then allows group comparisons to assess global ageing effects. In the present paper, the analyses compared the timing of EEG sensitivity to stimulus phase coherence (phase noise) between age groups.

Because we were interested in characterising the bottom-up response to faces, we sought to keep top-down factors constant. Therefore, we used a simple task in which subjects discriminate between two possible alternative pictures of faces, with image contrast far above detection threshold [60,63]. In addition to measurements of visual processing speed, we compared the topography of scalp data between age groups, based on the finding of an age-related decrease in hemispheric lateralisation associated with face processing [40,42].

## Methods

### Statistical analyses

Unless stated otherwise, statistical analyses were performed using a percentile bootstrap with alpha set to 0.05, and 5000 samples with replacement. In the text, square brackets indicate the boundaries of 95% confidence intervals (CI) constructed using this bootstrap technique [64]. Variances were compared using a percentile bootstrap in which the endpoints of the CI were adjusted to provide a better control over the probability of a type I error (see [64], pages 170-171).

In some situations, we used the shift function to compare entire distributions, instead of relying exclusively on point estimates like the mean or the median. In the shift function, the x-axis is the Harrell-Davis (hd) estimator of quantiles one to nine (see [64], pages 71-73, and 139-141). The y-axis is the difference, Delta, between the quantiles of the older and younger groups. Hence, the shift function represents how much the data from one group must be shifted to be comparable to the data from another group at each quantile. Group differences were estimated by a bootstrap procedure, and corrected for multiple comparisons such that the simultaneous probability coverage of the nine confidence intervals remains close to the nominal 0.05 alpha level (see [64], pages 151-155).

### Participants

A total of 15 younger and 18 older subjects participated in one behavioural experiment and one EEG experiment. Two younger subjects were excluded because of important muscle artefacts throughout the recordings. The remaining 13 younger subjects' mean age was 22 years (min = 19, max = 28, SD = 2.3); four were females; one subject was left-handed. Older subjects' mean age was 70 years (min = 62, max = 98, SD = 8.5); seven were females; all were right-handed. Older subjects were recruited from the Greater Hamilton Area community. Younger subjects were recruited from the McMaster University subject pool. All subjects received $10/hour for their participation and gave written informed consent. The McMaster University Research Ethics Board approved the research protocol.

All subjects reported that they did not have cataracts, macular degeneration, amblyopia, or any other visual pathology. Near and far Snellen acuities were measured with subjects wearing their regular optical correction. Younger adults had significantly better near decimal acuity (younger mean = 1.45, older mean = 0.95, difference = 0.5 [0.33 0.65], p = 0), and far decimal acuity (younger mean = 1.4, older mean = 1.08, difference = 0.32 [0.13 0.5], p = .0004) than older adults. In addition, older subjects completed the Mini-Mental State Examination (MMSE) to screen for age-related dementia. Median score was 29 (min = 26, max = 30), which corresponds to normative scores obtained in the age group 18-24 [65].

### Experimental design

**Figure 1:**
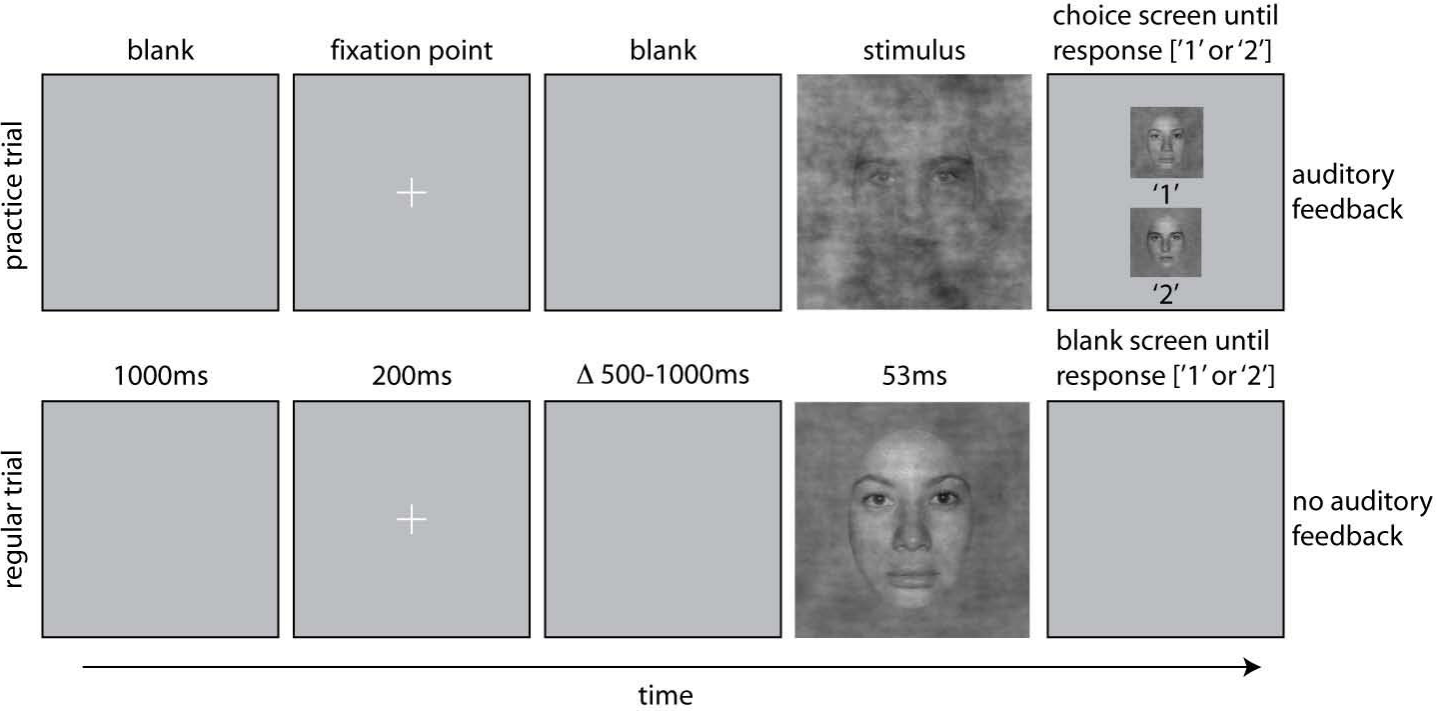
**Organisation of practice trials and regular trials in the two experimental sessions**. A trial started with a blank screen for 1000 ms, followed by the presentation of a fixation point for 200 ms. Then, after a random delay ranging from 500 to 1000 ms, a stimulus was presented for about 53 ms. During practice trials, a choice screen appeared immediately after the stimulus, showing the two targets of the experiment and their associated response keys. The screen stayed on until the subject's response, which was followed by auditory feedback, before the trial sequence resumed. During regular trials, a blank screen appeared immediately after the stimulus, and remained on until the subject's response. No feedback was provided during regular trials. Note that stimuli are not drawn to scale.

Younger and older observers were tested in two experimental sessions. The first day was a practice behavioural session; the second day consisted of both a behavioural task and simultaneous EEG recordings. On both days, subjects performed a one-interval, two alternative forced choice task between two faces. One pair of female faces and one pair of male faces were selected from a set of 10 faces used in previous experiments [66-68]. Each subject saw either two male or two female faces. On day 1, subjects were presented with 11 conditions along a noise-signal continuum (steps of 10% from 0 to 100%), while on day 2 only seven conditions were used (0, 30, 40, 50, 60, 70, 100% phase information, see below 'Stimuli'). Subjects sat in a dimly lit, sound-attenuated booth. Viewing distance was maintained at 90 cm with a chinrest. Stimuli were presented for about 53 ms (4 frames at 75 Hz) on a Sony Trinitron GDM-F520 monitor (800 × 600 pixels, width × height: 25° × 19° of visual angle). Subjects were given unlimited time to respond by pressing '1' or '2' on the numerical pad of the keyboard to indicate which face had been displayed (Figure 1). Subjects were told to emphasise response accuracy, not speed. The button-identity association was assigned randomly for each subject. The first day, the experiment consisted of 7 blocks of 132 trials (924 trials in total with 84 trials per level of phase coherence). The second day, there were 10 blocks of 84 trials (840 trials in total with 120 trials per level of phase coherence). Due to technical problems, one block was missing on day one for one older observer, and one block was missing on day two for another older observer. Within each block, there was an equal number of repetitions of each face and each phase coherence level. Each block was preceded by practice trials that allowed subjects to learn the stimulus-key association (20 in the practice session, and 10 in the EEG session). In a regular trial, a blank screen was presented for 1000 ms, followed by a small fixation cross (i.e., a 0.3 deg '+' in the middle of the screen) for 200 ms, after which another blank screen was presented for a random duration ranging from 500 to 1000 ms. Then a test stimulus was presented for 53 ms, followed by a blank screen that stayed on until subjects provided their response. Practice trials were very similar, except that immediately after stimulus presentation, a choice screen appeared that showed each face simultaneously, one above the other, with the corresponding label below each item. Auditory feedback was provided after the subject pressed a response key, with low- and high-pitched tones indicating incorrect and correct responses. Feedback was provided only during practice trials.

### Stimuli

**Figure 2:**
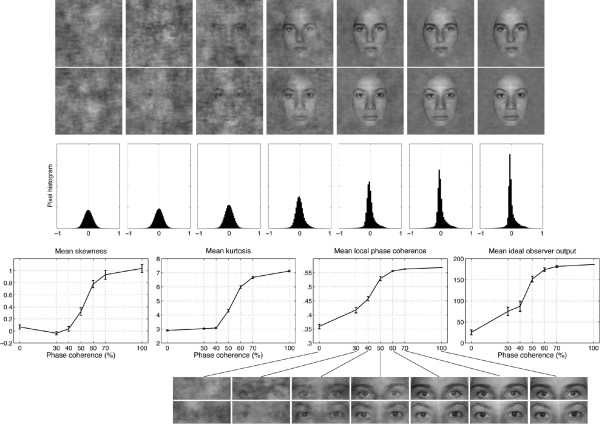
**Examples of stimuli used in the EEG experiment**. The first two rows show the 14 stimuli presented to one observer during the first block of the experiment. The observer discriminated the same two faces during the whole experiment. Phase coherence varied from 0% (left side) to 100% (right side) in six equal steps. Note different noise fields were mixed with the original image at each level of phase coherence, and therefore the task could not be performed based on the spatial characteristics of the noise. Histograms in the third row show the distribution of pixel contrasts averaged across all stimuli seen by this observer at each level of phase coherence. Starting with a Gaussian distribution (left side), the pixel histograms become increasingly skewed and kurtotic with increasing phase coherence (right side). Note that the y-axes on the histograms are all the same. This relationship is depicted in the last row, showing the mean skewness (left), and mean kurtosis (middle), as a function of phase coherence. Mean local phase coherence and mean ideal observer output were computed in the eye area depicted at the bottom of the figure. The error bars correspond to 95% confidence intervals.

Face stimuli were front-view greyscale photographs cropped within a common oval frame (6.2° × 4.4°) and pasted on a uniform 10° × 10° background (Figure 1). Models consented to have their pictures appear in publications. All stimuli had the same mean global amplitude spectrum and thus differed only in terms of global phase information, which carries most of the form information [61,62]. Six additional stimuli were constructed from each original face stimulus by manipulation of the phase spectrum, using the weighted mean phase technique (WMP, [57,60,69]), so that images were characterised by their percentage of phase coherence. This technique takes into account the directional nature of phase, assuring that phases are uniformly distributed after transformation. In comparison, a strict linear blend would lead to an over-representation of phases around 0°. Thus, WMP has the advantage over a linear blend technique to produce monotonic changes in third-order (skewness) and fourth-order (kurtosis) image statistics, as illustrated at the bottom part of Figure 2 and in [60,69]. For all stimuli, pixel contrasts ranged between -1 and 1 with a mean of 0. RMS contrast was constant across all levels of phase coherence. The amounts of phase coherence, skewness, and kurtosis, were included in a linear regression model of the EEG activity described below. In addition, we quantified two other image-based characteristics -- the output from an ideal observer and local phase coherence -- which are described in the next two sections.

### Ideal observer analysis

Previous research showed that human observers use a small area around the eyes and eyebrows to perform a face discrimination task like the one used in the present experiment [63]. We therefore performed an ideal observer analysis to measure the stimulus information available in this area. A unique area was defined to encompass the eyes and eyebrows of the four faces used in our experiment (Figure 2). For each observer, the two target stimuli, Face A and Face B, served as ideal templates that were cross-correlated (sum of pixel-by-pixel multiplications) with the stimuli shown at each noise level. For each image, the output of the ideal observer was the absolute difference between the convolution of that image with templates A and B.

### Local phase coherence

Kurtosis often is used as a measure of image sparseness and is correlated with the representation of phase structure: images with high levels of kurtosis generally contain local phase-congruent structures such as edges [70]. Local phase coherence is a measure of phase alignment across spatial frequencies at each pixel in an image that is independent of image contrast and luminance [71,72]. Previously, we argued that the EEG is sensitive to local elements such as edges [60]. Furthermore, subjects' behaviour in a natural scene classification task is correlated with measures of local image structure based on local phase coherence (e.g., lines, bars, edges) [73]. Therefore, the current study used an explicit measure of local phase coherence that was computed using a Matlab function provided by Peter Kovesi .

The function operates in three steps. First, local frequency information is extracted from an image by a bank of Gabor filters centred at 4 spatial frequencies (1.6, 3.5, 7.2, and 15.2 cycles per degree), and 6 orientations (0, 30, 60, 90, 120, and 150 degrees). Second, six maps of coherence are obtained for the six filter orientations. Each map is the same size as the image and contains values that range from 0 to 1, the minimum and maximum values for local phase coherence. Third, a single coherence map is obtained by taking the maximum moment of phase coherence across orientations. This final map has values in the range 0 to 0.62. The algorithm was applied to each image presented to our subjects. A summary value of local phase coherence was obtained for each image by taking the mean across the ten pixels with the highest values.

### Psychometric functions

The proportion of correct responses, *p*, at each level of stimulus phase coherence, *c*, was fit with a cumulative Weibull function,



where the scale parameter *α*, the shape parameter *β *that determines the slope of the curve in log-log coordinates, and the adjustment parameter δ, were estimated from the data.

The phase coherence *c *supporting a threshold performance of *Pc *= 0.75 correct was obtained using:



### EEG recording and preprocessing

EEG data were acquired with a 256-channel Geodesic Sensor Net (Electrical Geodesics Inc., Eugene, Oregon, [74]). Analog signal was digitized at 500 Hz and band-pass filtered between 0.1 Hz and 200 Hz. Impedances were kept below 50 kΩ. Subjects were asked to minimise blinking, head movement, and swallowing. Subjects were then given a description of the task. EEG data were referenced on-line to electrode Cz and re-referenced off-line to an average reference. The signal was then low-pass filtered at 30 Hz and bad channels removed, with no interpolation. The 30 Hz low-pass filter was justified by a previous study in which we showed that the differential activity evoked by faces and objects is contained mostly in a narrow 5-15 Hz band [75]. Baseline correction was performed using the 300 ms of pre-stimulus activity and data were epoched in the range -300 ms to 500 ms. Trials with abnormal activities were excluded based on a detection of extreme values, abnormal trends, and abnormal distributions, using EEGLAB functions [76,77]. The threshold for extreme values was ± 120 μV for all channels. An epoch was rejected for abnormal trend if it had a slope larger than 75 μV/epoch and a regression *R*^2 ^larger than 0.3. An epoch was rejected for abnormal distribution when its kurtosis fell outside five standard deviations of the kurtosis distribution for each single electrode or across all electrodes. All remaining trials were included in the analyses, whether they were associated with correct or incorrect behavioural responses. The average number of trials per subject was 590 [531 643] in younger observers, and 597 [546 643] in older observers, and did not differ significantly between groups (difference = -7 [-81 67], *p *= 0.86).

### EEG multiple linear regression analyses

Using a general linear regression approach, the single-trial EEG amplitude in μV was expressed, independently at each time point from 0 to 500 ms, using the following model:



Global phase coherence (φ_G_), kurtosis (γ_2_), local phase coherence (φ_L_), the output of an ideal observer (*I*), and skewness (γ_1_) were coded as continuous regressors, while the regressor for stimulus identity (S, i.e., Face A vs. Face B) was a categorical factor. The error term is (*ε*) and errors are assumed to be independent and identically distributed. The parameters of the model were z-scored before performing the fit. Therefore, regression coefficients (*β*_*i*_) were expressed in μV per SD of the regressor. The fit was performed at each electrode and each time point independently using the glmfit Matlab function, with a normal distribution.

In a previous study [60], we developed a similar model to understand the image factors driving the early EEG responses to complex visual objects, like faces. Results showed that the early EEG activity evoked by faces is modulated by global phase coherence, image kurtosis, and an interaction between global phase coherence and kurtosis. We interpreted this last interaction as reflecting sensitivity to local structure, such as edges. Here, we used a modified version of that model. Instead of relying solely on kurtosis as an indirect measure of local structure, the measures of local phase coherence and ideal observer output were used in an attempt to introduce parameters related explicitly to the local information used to perform the face discrimination task. Furthermore, the different parameters were submitted to a recursive orthogonalisation using the SPM 5 *spm_orth *function . This procedure ensures that the data are projected into regressor spaces that are orthogonal to each other, following a predetermined order in the fit that led to more stable estimates of the parameters across subjects. Based on our previous work, we orthogonalised the predictors in the order (1) global phase coherence, (2) kurtosis, (3) local phase coherence, (4) ideal observer output, (5) skewness and (6) stimulus identity. Skewness and stimulus identity were fitted last because in our previous experiment they did not contribute significantly to the fit. Importantly, different orders of the parameters would not affect the global model fit R^2 ^on which the data analyses were based.

Contrary to our previous study [60], the goal of the present paper was not to compare the relative contribution of the different regressors to the fit. Here, we used the time courses of the model fits across age groups (R^2^), to provide an estimate of age-related changes in visual processing speed. In addition, we assessed the topography of the effects by analysing the spatial extent of the R^2 ^clusters (spatial spread) and their hemispheric lateralisation (lateralisation index).

For each subject, we report the electrode at which the model provided the best fit (i.e., where R^2 ^was largest). The signal at that particular electrode was most sensitive to the structure of the image and therefore constitutes the most likely candidate for reflecting the activity of cortical sources sensitive to image information. In general, R^2 ^was the largest in a cluster of posterior electrodes that also exhibited large N170 responses to faces.

To estimate the statistical significance of the model fit, we computed R^2 ^bootstrap confidence intervals under the null hypothesis that all seven conditions were sampled by chance from the same population. This analysis was performed at the electrodes showing the best model fit. For each subject independently, we sampled with replacement individual trials from a pooled distribution of all seven conditions. Then, for each random sample, the linear regression model was fitted, and the R^2 ^and beta coefficients saved. Each sample consisted of the whole vector of time points because, unlike trials, time points are not independent from each other. The maximum R^2^, and the maximum absolute beta coefficients across time points were stored. This process was repeated 599 times and one-sided 95% confidence intervals of absolute values were computed. The maximum bootstrap statistics allowed a data driven control for multiple comparisons. This statistical analysis allowed us to determine the timing of EEG noise sensitivity in both groups.

### Topography analyses

We assessed age-related changes in ERP and model fit topographies by computing a hemispheric lateralisation index, and a clustering spatial spread measure of brain activity.

A lateralisation index was computed to estimate the degree of hemispheric lateralisation of scalp activity (ERP and model fit). First, scalp data normalised in the range [0 1] were interpolated and rendered in a 67 × 67 pixel image using the EEGLAB *topoplot *function. Second, the intensity of the pixels in the lower left and right quadrants, excluding the midline, were summed separately. These two quadrants contain the electrodes at which visual brain activity to faces typically is recorded. Finally, the lateralisation index was computed as the ratio (Σ_left _- Σ_right_)/(Σ_left _+ Σ_right_).

A spatial spread measure was used to estimate the degree of clustering of scalp data around the electrode showing the maximum activity. First, scalp data normalised in the range [0 1] were interpolated and rendered in a 67 × 67 pixel image using the EEGLAB *topoplot *function. Second, the pixel image was centred (mean set to zero), and squared. Third, pixel intensities *V*_*i *_were weighted by their distance *D*_*i *_from the pixel with maximum intensity, and summed. Finally, spatial spread was computed as the ratio:



## Results

### Behavioural results

**Figure 3:**
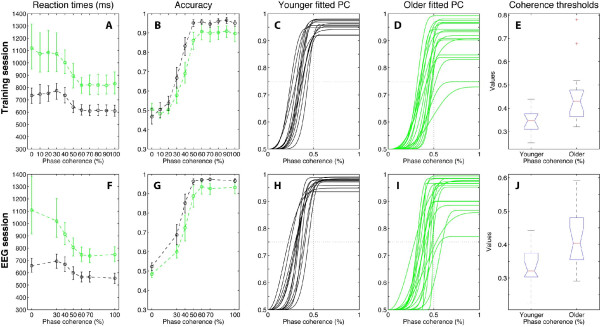
**Behavioural results in the training (upper row) and the EEG (lower row) sessions**. Green = older, black = younger. Error bars = 95% confidence intervals. The third column shows the individual percent corrects (PC) (averaged across subjects in column two) fitted by cumulated Weibull functions. Stimulus phase coherences necessary to reach a threshold performance of 75% correct were obtained from the Weibull fits, and are shown as boxplots for younger and older observers in the last column. In a boxplot, the red line indicates the median. The blue box extends from the upper to the lower quartile values. The whiskers show the most extreme points that are within 1.5 times the inter-quartile range. A red plus is an outlier. The notch in the blue box corresponds to a robust estimate of the median confidence interval. Non-overlapping notches indicate that medians differ with 95% confidence.

The group means of the individual median reaction times were, overall, longer for older subjects, and were more influenced by phase coherence than in younger subjects (Figure 3A, F). Proportion correct was significantly higher for younger subjects than for older subjects in both the training session and in the EEG session. Two older subjects had undefined thresholds in the training session, because their maximum performance did not reach 75% correct (Figure 3D). Extrapolated thresholds were estimated to be 0.78 and 0.68 (marked as outliers in Figure 3E). These two subjects improved in the EEG session and their thresholds went down to 0.57 and 0.59 (Figure 3I-J).

In the EEG session, mean maximum percent correct was significantly lower in older (90.5% [86.8 93.7]) than younger subjects (95.7% [94.6 96.7]; difference = 5.2% [1.7 8.9], *p *= 0.0012). However, a shift function revealed that the two groups differed significantly only in the first decile, which can be explained by the strong overlap between the two groups and the higher variance in the older group (difference in variance of maximum percent correct = -0.0056 [-0.0105-0.0008], *p *= 0). Concretely, the shift function results mean that response accuracy in the worst older subjects was significantly lower than accuracy in the worst younger subjects, but otherwise the two groups were similar. The mean stimulus phase coherence required to achieve 75% correct was significantly higher in older (42.1% [38.4 46.1]) than younger subjects (33.1% [30 36.3]; difference = -9 [-1.4-4], *p *= 0.0008). The distributions of thresholds in the two age groups overlapped considerably: a shift function analysis revealed that the two groups differed significantly only in the ninth decile. However, the variances of the two groups did not differ significantly (variance difference = -0.0037 [-0.0103 0.0026], *p *= 0.1867). This result implies that only a few older subjects drove the overall age difference in threshold.

### ERP results: time course and N170 analyses

A single-trial regression model was applied at each time point and electrode. The mean maximum value of R^2 ^for older observers (34% [29 40]) and younger observers (39% [33 45]) did not differ significantly (mean difference = -4% [-12 4 ], p = 0.2848), indicating a similar model fit for all subjects. For each subject, we selected the electrode at which the maximum R^2 ^was found. R^2 ^was largest in a cluster of posterior electrodes that also exhibited large N170 responses to faces. We report analyses first for the N170, measured at the electrode with maximum R^2^, then for the model fit.

**Figure 4:**
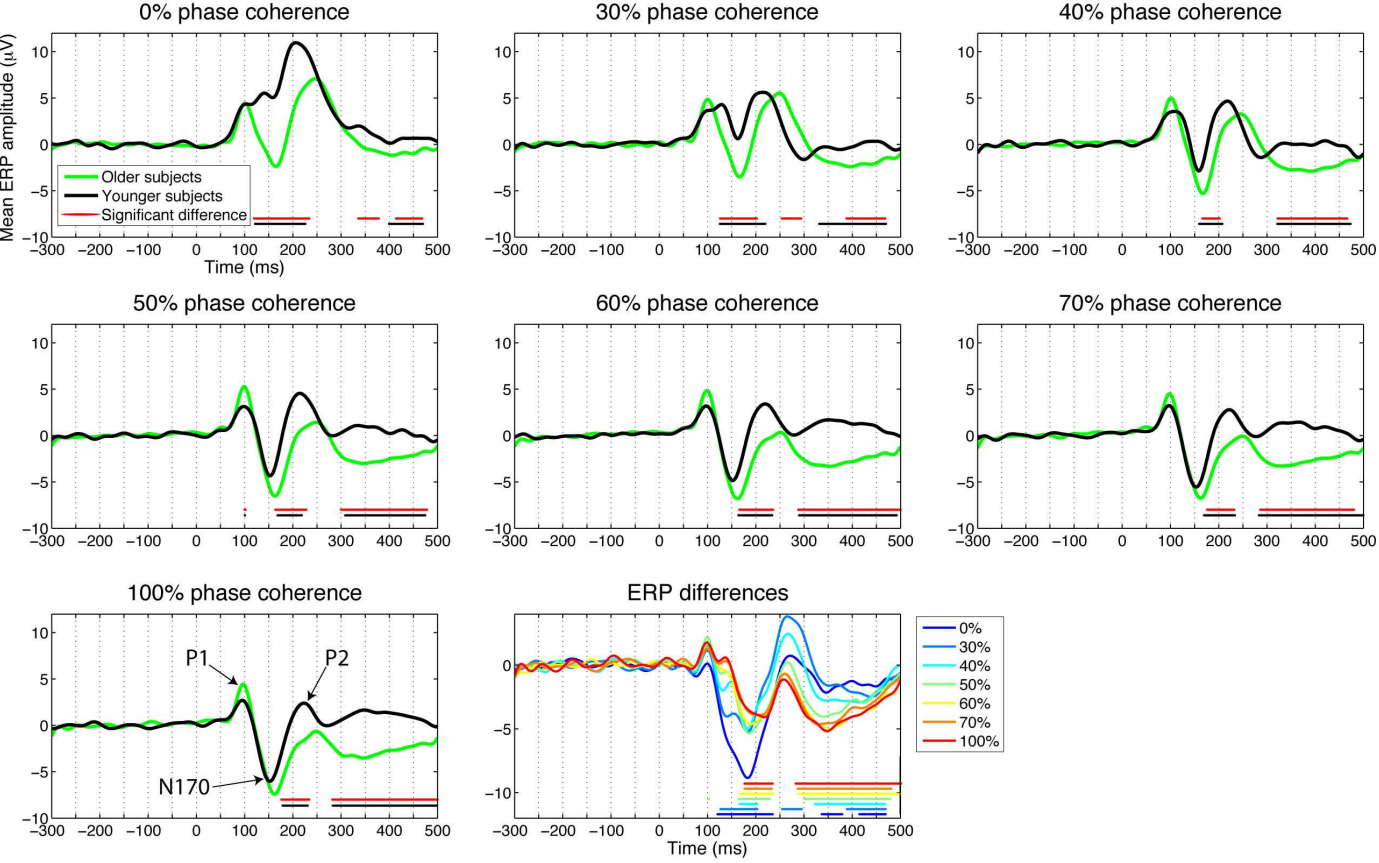
**Running tests of mean ERP group differences, at the electrode presenting the best model fit, between younger (black) and older (green) observers**. For the different levels of phase coherence, in the different subplots, horizontal red lines indicate time points at which a difference was significant (95% percentile bootstrap). The horizontal black lines show the results of the same analysis, performed on the modelled ERP instead of the original ERP data. The lower right subplot directly compares the differences across the seven levels of phase coherence. In this subplot, the horizontal lines are colour coded to represent time points of significant effects in the different conditions.

**Figure 5:**
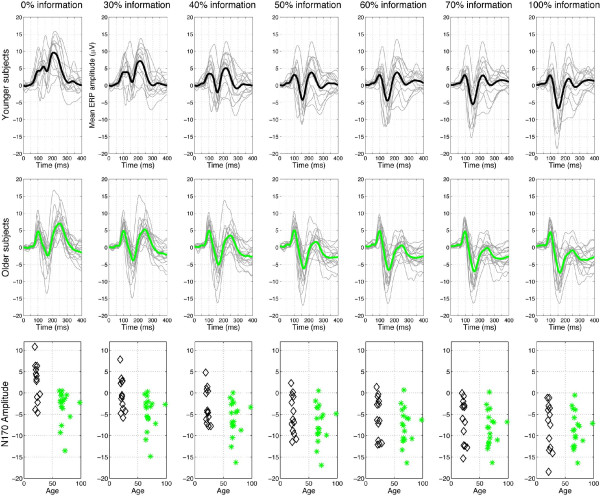
**Mean ERP for old (top row) and young (middle row) observers at each level of phase coherence (% information)**. ERP from individual subjects are depicted in thin grey lines, with the mean across observers in thick black (younger) and green (older) lines. Note the very large N170 recorded in the noisy conditions for older observers only (middle left). In the bottom row, N170 amplitudes are shown for each subject as a function of age at each level of phase coherence. Black triangles = younger subjects, green stars = older subjects.

Figure 4 shows the mean ERP for older and younger observers at each level of phase coherence. ERP differences between older and younger observers were first tested systematically over time at each level of phase coherence, taking for each subject the electrode showing the strongest R^2^. The most striking and earliest group differences in terms of mean or median ERPs occurred in the time window of the N170, particularly in the conditions with the lowest percentage of stimulus information (0% phase coherence): In this condition, older, but not younger, subjects exhibited a pronounced N170 (Figures 4 and 5). These group differences were delayed and became weaker with increasing stimulus phase coherence. Identical results were obtained when the analyses were performed on the modelled ERPs, which suggests that the model captured the essential aspects of the EEG associated with variations in image structure imposed by the noise manipulation. Comparisons of average higher-order EEG signal statistics (variance, skewness, and kurtosis) did not reveal any significant difference between the two age groups at any level of phase coherence.

The N170 peak latency and amplitude were also measured for each subject at the electrode showing the best model fit (maximum R^2^). The N170 latency on average was delayed in older subjects compared to younger subjects, suggesting a delay in visual processing speed in older subjects. The mean difference between groups ranged from a minimum of 8 ms in the 0% phase coherence condition ([-5 22], p = 0.2164), to a maximum of 13 ms in the 60% phase coherence condition ([3 23], p = 0.0096), and was 9 ms in the 100% phase coherence condition ([-1 19], p = 0.0728). In addition to the latency differences, significant amplitude differences were observed: The main result was that older observers had a surprisingly strong N170 in response to noise (0% phase information), a pattern clearly different from the one observed in younger observers (statistics on means: younger = 2.4 [0.2 4.8]; older = -3.2 [-4.9-1.7]; difference = 5.6 [2.8 8.6], p = 0.0004). Interestingly, this age difference decreased with increasing phase information (Figures 4 and 5).

There was no evidence that older subjects who exhibited stronger N170 amplitude to noise performed worse than the others on our behavioural measures. We tested a model in which we tried to predict the behavioural thresholds from the difference between N170 amplitudes in the 0% and 100% conditions. The model also included an age group categorical variable, and an interaction term between the categorical and continuous variables. Our rationale for testing this model was that behavioural thresholds might be related to the sensitivity of the N170 to stimulus noise. The model provided a significant fit of the data (*F *= 3.8, *p *= 0.0216, R^2 ^= 30%). The age group effect was significant (*p *= 0.0029), which is consistent with our observation of lower thresholds in younger subjects. However, the N170 amplitude differences variable (*p *= 0.3348), and the interaction term (*p *= 0.9808) failed to predict the behavioural thresholds significantly. Thus, in both younger and older subjects, there was no evidence of a significant relationship between the noise-face N170 differential amplitude and subjects' behavioural thresholds.

Finally, we performed a topography analysis of the N170. Topographic maps of modelled data were obtained in individual subjects at the latency of their N170, which was measured at the electrode yielding the highest R^2^. For both groups of subjects, the N170 lateralisation index (see Methods) revealed a stronger signal over right than left hemisphere electrodes in the 0% and 100% phase coherence conditions, but the lateralisation index did not differ significantly between the two age groups (e.g., in the 100% condition, difference = 0.05 [-0.04 0.15], *p *= 0.2740).

In sum, the ERP analyses revealed a striking difference between the two age groups in the time window of the N170 at the 0% phase coherence level. This result, and the fact that the N170 did not differ between groups at 100% phase coherence level, implies that manipulating phase information had a greater effect on ERPs from younger observers. Furthermore, this result was obtained even when the N170 amplitude was normalized for each subject, which means that the group difference in phase sensitivity shown in Figure 5 was not due to differences in absolute signal amplitude. This observation also was confirmed by the results of the linear regression analysis described in the next section.

### ERP results: general linear regression analyses

In this section we describe analyses of topography of the model fit, onset and time course of the model fit, and finally analyses of the relationship between EEG measures and other measures (behaviour, N170).

We performed a topography analysis that was independent of ERP components by computing the lateralisation index on the R^2 ^maps at the latency of the maximum R^2 ^for each subject. Thus, in this analysis, maps at different time points are used for different subjects. Similarly to the N170 results, both age groups tended to be right lateralised, and the two age groups did not differ significantly from each other (difference = -15 [-185 185], *p *= 0.8356). The two age groups also did not differ in terms of spatial spread (difference = 4 [-35 48], *p *= 0.8708). Overall, these topography analyses suggest that noise sensitivity tended to be stronger over the right hemisphere in both age groups, with no evidence for a significant change in lateralisation or spatial extent of the sensitivity to noise with age.

**Figure 6:**
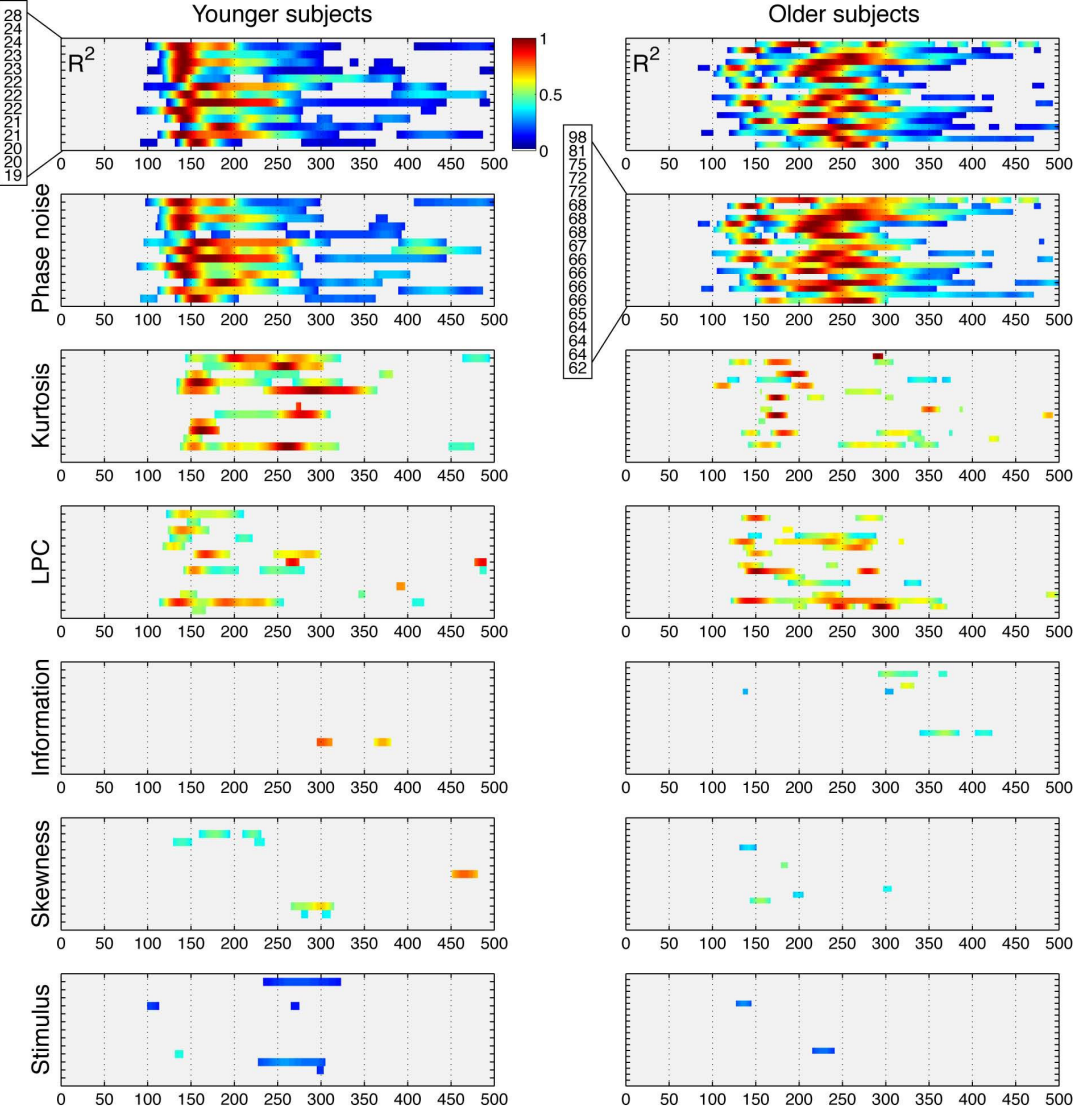
**Normalised R^2 ^and beta coefficients for the two age groups**. R^2 ^were normalised independently in each subject by the maximum across time points. Beta coefficients were normalised independently in each subject by the maximum across time points and beta coefficients. In each subplot, subjects were sorted from the oldest (top) to the youngest (bottom).

Although both age groups tended to have a right hemisphere lateralisation of the EEG noise sensitivity, they differed in terms of timing (Figure 6, top row). In older subjects, the function describing the time-course of the model R^2 ^had a delayed onset and a delayed peak. Older subjects also had more heterogeneous results than younger subjects. In both age groups, modulations of evoked activity were due mainly to phase coherence, with the additional contribution of kurtosis and local phase coherence, and virtually no contribution from other factors (Figure 6). The similarity in EEG sensitivity to the different image statistics factors suggests that early visual activity was modulated by the same stimulus parameters in younger and older subjects. The differences observed between the two age groups thus reflect mostly differences in the timing of the EEG sensitivity to the same parameters (i.e., visual processing speed).

The model onset time was defined as the earliest time point at which R^2 ^was significantly larger than expected by chance based on a bootstrap simulation (see Methods). The mean model onset time was 110 ms [102 117] in younger adults and 129 ms [115 145] in older adults: evoked responses in younger adults exhibited sensitivity to image structure significantly earlier than responses in older adults (-20 ms [-37-4], *p *= 0.0144). However, there also was evidence of a substantial overlap between the two groups as no differences were observed when using the median onset time (112 ms [98 118] in younger adults, and 119 ms [111 149] in older adults, median difference of -7 ms [-37 2], *p *= 0.1020). The variance of the model onset time was greater in older than younger subjects (variance difference between the two groups = -887 [-2062-46], *p *= 0.01), and the effect on the mean was driven by extreme values in older adults to which the median was less sensitive.

**Figure 7:**
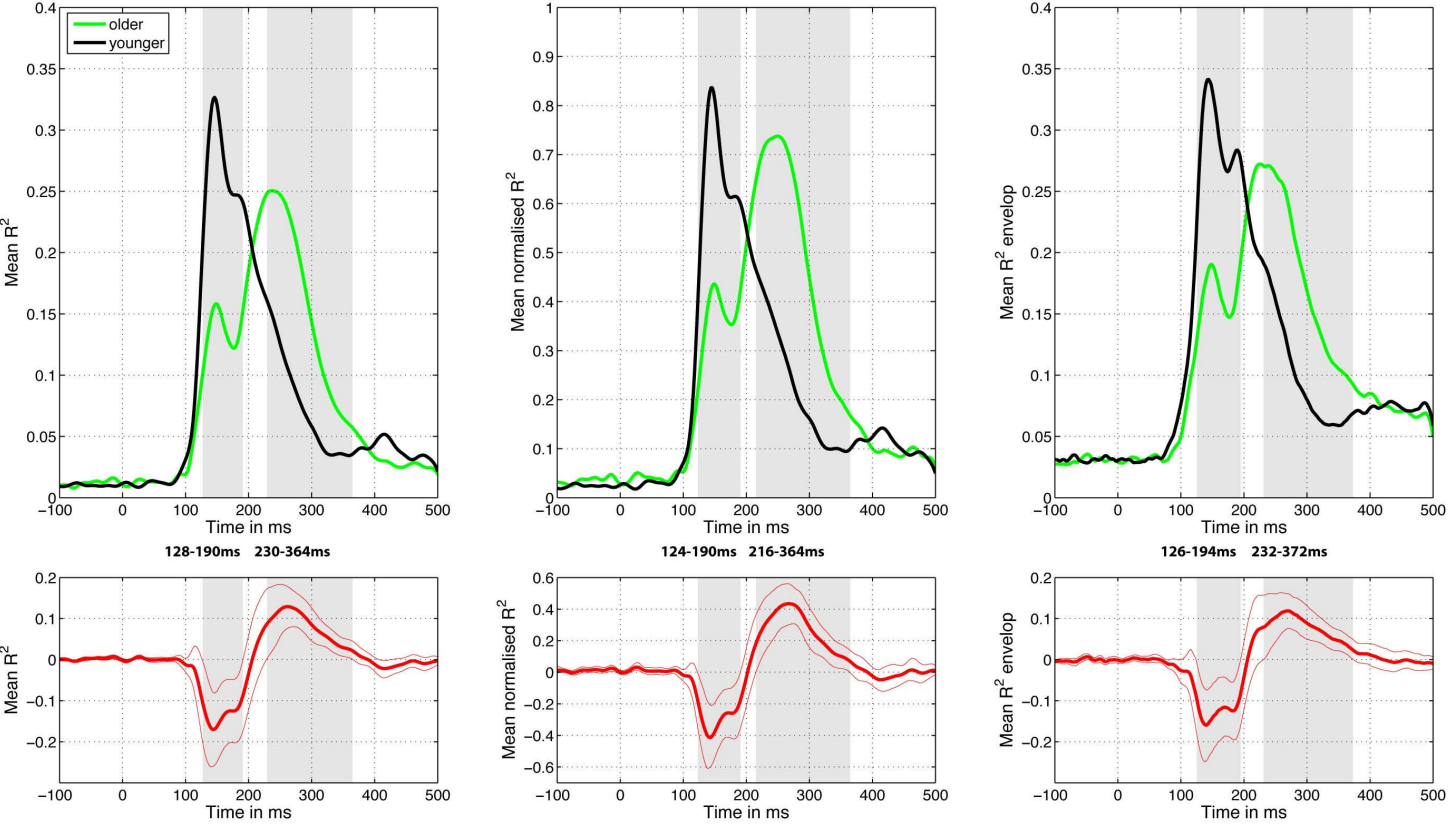
**Mean, normalized and envelop R^2 ^results**. Mean R^2 ^for younger (black) and older (green) subjects were compared. The difference between the two groups is plotted in thick red lines, with the 95% confidence interval in thin red lines. When the difference does not contain zero, the difference is significant, which is indicated by the grey areas. The boundaries of the time windows in which significant differences were observed are indicated between the two graphs in each column.

In Figure 7, R^2 ^time courses are illustrated and compared independently at each time point. In some respects R^2 ^time courses are more informative than raw ERPs because they eliminate effects caused by group differences in absolute response amplitude and, furthermore, provide a response metric that is based on the visual system's sensitivity to image information. Consistent with our previous findings, the R^2 ^functions measured in younger subjects had one peak in the time-window 100-200 ms [60]. In sharp contrast, R^2 ^functions for older subjects had two peaks: one smaller peak in the 128-190 ms time window, and a second larger peak in a later time window of 230-364 ms (Figure 7, left). Nearly identical findings were obtained if the R^2 ^functions were first normalized to have a peak of 1 for each subject (Figure 7, middle), or if the analysis was performed on an envelope R^2 ^function, which was computed by taking the maximum R^2 ^value across all electrodes independently at each time point (Figure 7, right). Figure 7 shows that sensitivity to image information grows more slowly in older subjects. In addition, Figure 7 indicates that the time course of the model fit differs qualitatively across age groups, being almost monophasic in younger subjects and biphasic in older subjects. Also, in Figure 7, there is a shoulder on the right side of the peak for younger subjects, slightly before 200 ms. This shoulder corresponds to 3 subjects with a broader and delayed first peak, and 5 subjects showing a second, weaker peak in that time window (Figure 6 top left). This second peak in some younger subjects might correspond to a significantly earlier version of the second peak evident in the older data.

**Figure 8:**
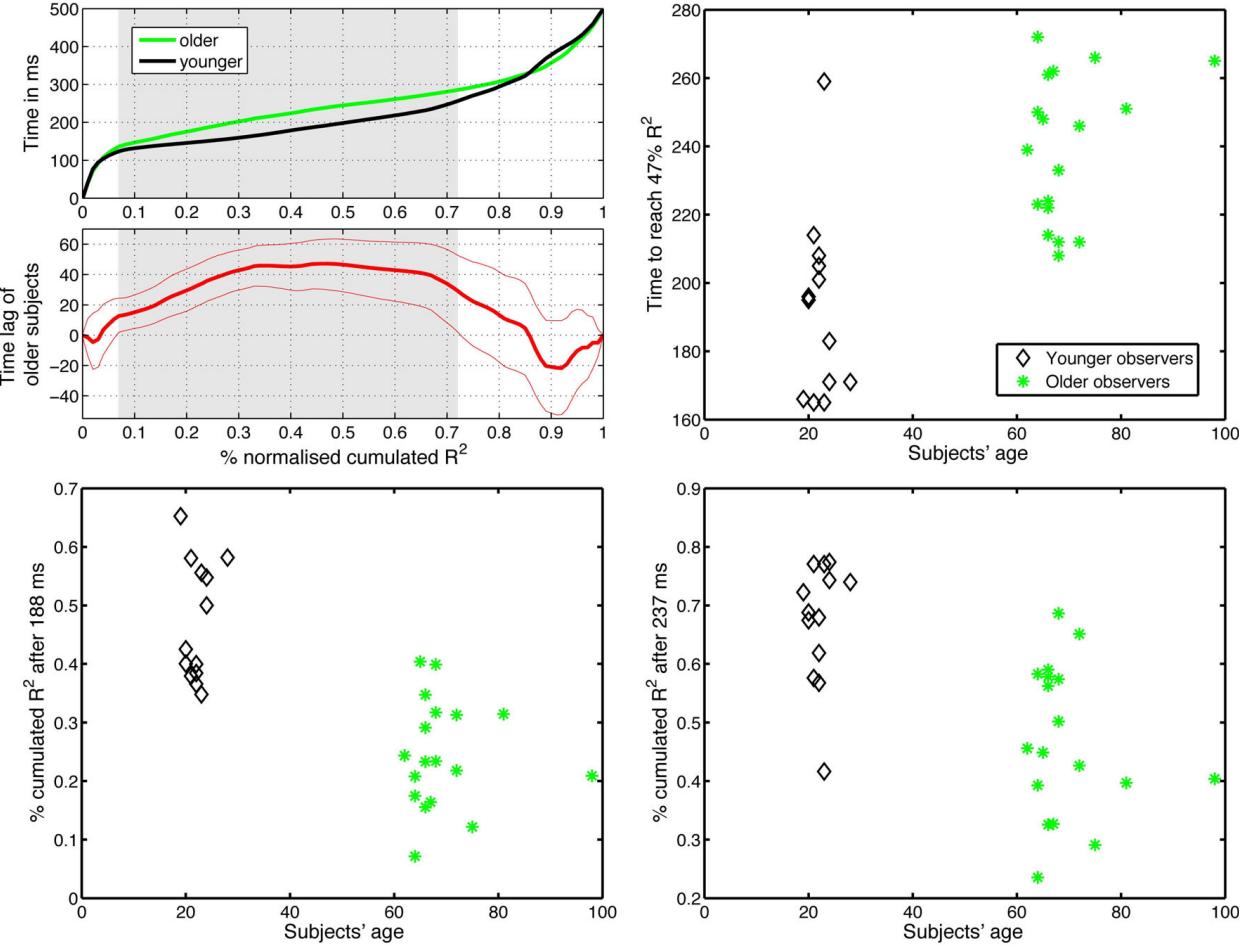
**Processing time thresholds**. The upper left quadrant shows the mean time in ms (y axis) necessary to reach a given percentage of the normalised cumulated R^2 ^time course (x axis) for younger (black) and older (green) observers. The difference between the two groups is plotted in thick red lines, with the 95% confidence interval in thin red lines. When the difference does not contain zero, the difference is significant, which is indicated by the grey areas. After the younger subjects had accumulated 47% of their R^2 ^distribution, they leaded the older group by 47 ms. The time necessary to reach 47% is shown in the upper right quadrant for individual subjects. The percentage of the R^2 ^distribution accumulated after 188 ms and 237 ms are indicated in the lower quadrants. See text for explanations.

The time-course of the model fit illustrated in Figure 7 indicated that, in older adults, the EEG signal is sensitive to the image structure during a longer period of time, spread out over two peaks, than in younger adults. To compare the R^2 ^functions at each time point in the two groups, independently of whether they presented one or two sensitivity peaks, we looked at the data from a different perspective. Instead of determining at each time point differences between mean group R^2^, we determined how long it took a subject to reach a certain R^2 ^level. To avoid biases by absolute R^2 ^values, we computed the percentage of cumulated normalised R^2 ^for each subject. In detail, for each subject, we computed a cumulated sum of the R^2 ^in the time window 0-500 ms. That cumulated sum was then normalised between 0 and 1 (i.e., it had a value of 0 at stimulus onset, and a value of 1 at 500 ms after stimulus onset). Then, we computed the time necessary to reach each of 101 steps between 0 and 1. At each step, the values were extrapolated using a cubic spline interpolation. The results, averaged across subjects, are presented in the upper left quadrant of Figure 8.

The earliest significant difference between the two age groups emerged when 7% of R^2 ^had been accumulated, which corresponded to a latency of 122 ms in younger observers and 129 ms in older subjects. The maximum time difference between the two age groups was 47 ms [30 63 ms] (Figure 8, upper right quadrant). This difference was observed when younger subjects had accumulated an average of 47% [38 56] of their R^2^, which was reached at a latency of 188 ms (Figure 8, lower left quadrant). At that latency, older observers had accumulated only 25% [19 31] of their R^2 ^distribution (the difference between the group means was 23% [16 29], *p *= 0), and they reached 47% [40 57] at 237 ms (Figure 8, lower right quadrant). At this latency, younger observers reached 67% [62 74], which led to a significant difference between the two groups of 20% [12 28], *p *= 0. Thus, in terms of the pace of accumulation of R^2^, younger subjects outperformed older subjects as early as 122 ms after stimulus onset, and had the largest time lead 66 ms later, at 188 ms.

Finally, we performed a general linear regression across younger and older subjects in which we tried to predict the cumulated R^2 ^thresholds. The linear regression model included the categorical predictor age group and continuous predictors close Snellen acuity, far Snellen acuity, and contrast sensitivity. The overall model provided a significant fit (*F *= 8.2512, *p *= 0.0002, R^2 ^= 56%), but only the age group variable was significant (*p *= 0.0199, all other betas *p *> 0.15). Similarly, none of these factors predicted model onset times. Thus, a simple factor like increased blur (decreased visual acuity) is not likely to explain the increased variance and delayed timing in the older group. In a last comparison, we found no significant relationship between the times to reach 47% cumulated R^2 ^and the 75% correct behavioural thresholds.

## Discussion

The goal of the present study was to quantify age-related changes in the time-course of visual processing during the perception of complex objects, faces, while subjects were engaged in a simple discrimination task. We found evidence of both quantitative (slowing of visual processing) and qualitative (larger N170 to noise, biphasic R^2 ^function) changes in EEG patterns, in conjunction with poorer behavioural performance in older adults. First, we discuss the behavioural results, and their relationship to EEG data, before focusing on the potential origin of the age-related changes in the visual processing time-course.

The behavioural results, showing higher 75% correct thresholds, and lower maximum performance with age, suggest that older participants were not able to extract as much information from the stimuli as the younger participants. This result might indicate that older subjects are, on average, less able to encode information in the global phase spectrum, a topic that has not been studied so far to our knowledge. However, it is important to consider that the behavioural age-related decrease in performance was a group effect, and that there was substantial overlap between individuals in the two groups. The same conclusion applies to the EEG results, which revealed not only an age group effect in processing speed, but also a large variance among older adults, that could not be accounted for by chronological age alone. This resonates with several observations made in the ageing literature [1,78]. Our own ageing results, and the overwhelming majority of results published so far, thus demonstrate that we have to go beyond the cognitive neuroscience of the average brain and to look at inter-individual differences to understand the ageing brain.

Using the model fit (R^2^), we observed that older observers have a delayed EEG sensitivity to stimulus structure, with R^2 ^differences in the time windows 124-194 ms and 238-374 ms, and maximum cumulative R^2^difference of 47 ms. Some of these observers also had a delayed model onset time (i.e., the time when R^2 ^first differed significantly from zero). Despite these age-related slowing effects, we were unable to find any fine-grained relationship between face processing speed and behavioural performance: older subjects showing strong delayed phase sensitivity did not perform worse than average. Future experiments should aim at dissociating age-related changes in EEG that are correlated with behaviour, from age-related changes in EEG that are not correlated with behavioural differences. In older subjects, it might be that a delayed but pronounced sensitivity to phase is sufficient to perform our face discrimination task. After all, a delayed extraction of phase information should not affect behaviour, which is a measure of the end process only, particularly in a non-speeded task like the one we employed. This hypothesis is consistent with the idea that changes in visual processing speed do not imply that less information is extracted from the stimulus because the two can be dissociated [34]. If we consider that behavioural outputs also take into account decision-making stages, it might be that older adults had more difficulty basing their decision on more limited evidence in our task, because of the rather brief presentation duration used (53 ms). In other words, older adults might need more temporal integration to achieve the same level of performance as younger adults, similarly to what has been demonstrated recently for motion perception [10]. Thus, in older observers, the stimulus analysis might start at about the same latency (being only slightly delayed in some but not all observers), but because of a slower accrual of stimulus information, behaviour would be more strongly affected by brief stimulus presentation. The age-related slowing in visual processing observed in the EEG data might have different origins that we will now discuss.

### Age-related processing speed decrease in the face cortical network?

We found an age-related decrease in processing speed that emerged after about 120 ms, increased over time, and then declined to near zero beyond 300 ms after stimulus onset. The largest age differences occurred in the time-windows of the P1 and N170 components, which are typically localised in extra-striate cortex, and more specifically in cortical areas of the face network [27,52,53]. In addition, the phase coherence manipulation we employed has been shown to have the strongest effects in higher-order cortical areas involved in object and face processing (see discussion in [60]). There also is evidence that the same cortical areas are recruited in both younger and older adults across different noise levels while subjects are engaged in different face categorisation tasks [16,79]. For these reasons, it seems reasonable to assume that the EEG effects we measured were generated predominantly in cortical areas sensitive to faces and objects.

Interestingly, the absence of earlier effects in our study and in previous ones is at odd with results showing age-related changes in visual processing speed in monkey areas V1 and V2 [4], as well as in rats' primary visual cortex [80]. However, it is not clear whether the biased sample of cells in monkey studies [81] can be predictive of EEG scalp data, which reflects local field potentials rather than single-unit activity [82]. Along the same lines, discrepancies have been reported between single-unit results and psychophysical results [83]. Thus, it remains to be determined which part of the neuronal response a decision is based upon while making comparisons across species and scales. Finally, the lack of early effect in our study could be due to stimuli not optimal to trigger differential activity from early visual areas. Indeed, natural images like faces and pink noise might have similar neuronal signatures in V1 because they have the same 1/f, 1/f^2 ^amplitude spectrum, to which V1 response properties might be particularly tuned (see discussion in [60]). Hence, contrasting responses to pink noise and white noise, for instance, might help isolate V1 specific responses.

At least one of the nodes of the face network potentially generating the N170, the fusiform gyrus [51,53,84], undergoes a loss of response tuning with age. This was demonstrated by fMRI results showing reduced BOLD difference between faces and pink noise in older adults compared to younger adults in the ventral visual cortex [85]. The same fMRI study, and a more recent one [86], also reported a dedifferentiation of the BOLD response for pictures of faces, words, places, chairs, and houses, whereby, in voxels with a preferential, larger response to one category, the response to other categories increased with age. These recent fMRI results are in line with earlier studies showing that the same areas are recruited during face perception in older and younger adults, but that in older adults there might be a drop in efficiency [16,79]. The more recent fMRI results suggest that the sensory dedifferentiation is not limited to faces, but applies to other object categories as well. Thus, although the fMRI results and the present results cannot be compared directly because EEG does not have the spatial resolution to distinguish such fine categorical responses, our results suggest that a form of age-related loss in higher-order signal sensitivity takes place in the extra-striate visual cortex, in the time-window 100-300 ms. In particular, our results suggest that the N170 becomes less face-sensitive with age. It will be interesting to determine if processing speed is similarly affected for categories other than faces in future EEG experiments.

### Origin of the age-related delayed sensitivity to image structure

The origin of the reduced face sensitivity of the N170 in older adults remains to be explained. A simple explanation like neuronal loss seems unlikely because, although there is a decrease in grey matter density with age in the extra-striate visual cortex [87,88], the number of cortical neurons in the visual cortex appears to be stable across the life-span [89]. However, other changes take place, such as an age-related decrease in GABA mediated lateral inhibition that leads to reduced neuronal response tuning in early visual cortical areas [9,19,24,90,91]. Interestingly, the tuning of neurons sensitive to objects in the inferior-temporal cortex depends to some extent on lateral inhibition [92,93]. Thus, it is plausible that the degradation of GABA mediated inhibition with age is responsible for a progressive loss of categorical tuning in cortical areas mediating object processing. This dedifferentiation of neuronal responses would lead to noisier outputs, and to longer processing times following a model of visual processing by accumulation of evidence [94,95]. Such lack of lateral inhibition in object cortical areas might explain the larger response to pink noise we observed in older subjects. Hence, it will be interesting to determine if that effect can be generalised across spectral categories, for instance by testing older subjects with pictures of stimuli from different categories, and the associated phase randomised images.

Even if age-related changes in local inhibition might explain the delayed face sensitivity in our results, this explanation is rather speculative at this point and further research is needed. Already, we note that the idea of an age-related increase in neuronal noise would predict a lower model fit R^2 ^in older subjects, which was not the case. Nevertheless, it could be that only some part of the EEG evoked response is affected by increased neuronal noise, possibly in the time window of the N170, but that later periods of activity, maybe reflecting the contribution of other cortical areas, are less affected by such changes. It is also worth pointing out results from other EEG studies that support the existence of an age-related increase in evoked activity, although very few, to our knowledge, have contrasted the responses to different categories of stimuli [31,32,42,43,96]. However, some studies have reported opposite effects, for instance [37].

The literature on ageing and face processing also has suggested the existence of a change in the hemispheric distribution of activity, from right lateralised to more bilateral activity in older adults [40,42,79]. We found no evidence of a change in hemispheric lateralisation with age in our experiment. Ageing has also been associated with more widespread distribution of EEG on the scalp, which could reflect a larger recruitment of brain areas because of reduced cortical inhibition [96]. However, our analyses revealed no significant differences in the spatial extent of EEG sensitivity to phase coherence with age.

In addition to the suggested changes in local inhibition, and in the distribution of cortical activity, it has been proposed that ageing leads to a decrease in the number of synapses and nerve fibres, and an alteration of the myelin of cortical neurons [20,21,97]. In particular, myelin sheath alterations have been observed in the primary visual cortex [20,98]. It has been suggested that the alteration in myelin sheath might lead to delayed conduction rates along the affected nerve fibres, which in turn might affect the timing of neuronal events, thus leading to cognitive decline [18,98]. These changes in myelin sheath suggest a rather distributed origin of age-related decay, whereby communications both within and between cortical areas might be perturbed.

As noted above, the same network of brain areas is activated in younger and older observers during face perception [16,79]. However, when task difficulty increases, for instance because faces are degraded, older observers rely more on prefrontal areas, suggesting that, with age, there is an over recruitment of frontal activity to compensate for poorer performance of the sensory systems [3,99]. There also is evidence that the frontal cortices tend to decrease in volume the most with age [88]. In addition, with ageing, there is a decreased number of synapses and an alteration of the myelin sheath in the prefrontal cortex [100]. These changes in the prefrontal cortex might mediate a large part of the cognitive decline with age. More specifically, the age-related delay in visual activity observed in the present experiment might reflect a lack of proper top-down control, in which the prefrontal cortex controls the flow of information in the ventral visual cortex [38,101-103]. In a similar vein, the age-related delay could reflect a strategy difference from older adults who struggled to discriminate faces in noisy conditions and therefore treated each stimulus as being a face. Finally, because the difference we observed between younger and older observers built up over time, rather than showing a fixed lag between the two age groups, it might reflect the interaction between prefrontal and ventral cortices during the first half second of face processing [39,104-106]. Hence, to finish on a more optimistic note, the age-related processing speed delay, rather than reflecting a lack of top-down control in older subjects, could reflect a difference in strategy.

## Conclusion

Very few studies have attempted to measure the effect of ageing on the time-course of visual processing in response to complex stimuli like faces. We bring here several innovations to tackle that question, using a single-trial linear regression approach that does not make assumptions about a priori time windows or components of interest. We confirmed previous findings of an age-related delay in face processing speed in the time window of the N170. We extended these findings by precisely quantifying the processing speed delay as indexed by the time course of the EEG sensitivity to image structure. Our results suggest that stronger dissociations between younger and older observers can be obtained using noise textures rather than easily discriminable stimuli. The use of these stimulus classes also enabled us to show that the N170 response was less face-sensitive in older observers. Particularly noteworthy was the significant overlap between age groups observed for behavioural results, and some of the EEG results, a result too often ignored in other studies. Our discussion points to two plausible sources of the age-related delay. First, reduced GABA mediated lateral inhibition, causing a dedifferentiation of the neuronal responses in face sensitive areas. Second, changes in white matter affecting cortical communications within face processing areas, between face processing areas and frontal areas, or both. Further experiments will be necessary to determine whether these two complementary explanations can address our results. Finally, it will be important to assess the generalizability of the age effects on processing speed across tasks and stimulus categories, particularly to determine if results from a simple face discrimination task could be predictive of impairments in more realistic situations.

## Authors' contributions

GAR, JSH, PJB, and ABS designed the study. GAR and JSH collected the data. GAR conducted the analyses and wrote the manuscript. CRP and CMG helped analyse the data. JSH, CRP, CMG, PJB, ABS helped revise the manuscript. All authors read and approved the final manuscript.
